# Supplementary figures and images for: Genome-Wide Analysis of the bHLH Gene Family in Loropetalum chinense var. rubrum: Identification, Classification, Evolution, and Diversity of Expression Patterns under Cultivation
Source: Plants (Basel). 2023 Sep 26;12(19):3392. doi: 10.3390/plants12193392 (PMC10574408; doi:10.3390/plants12193392)

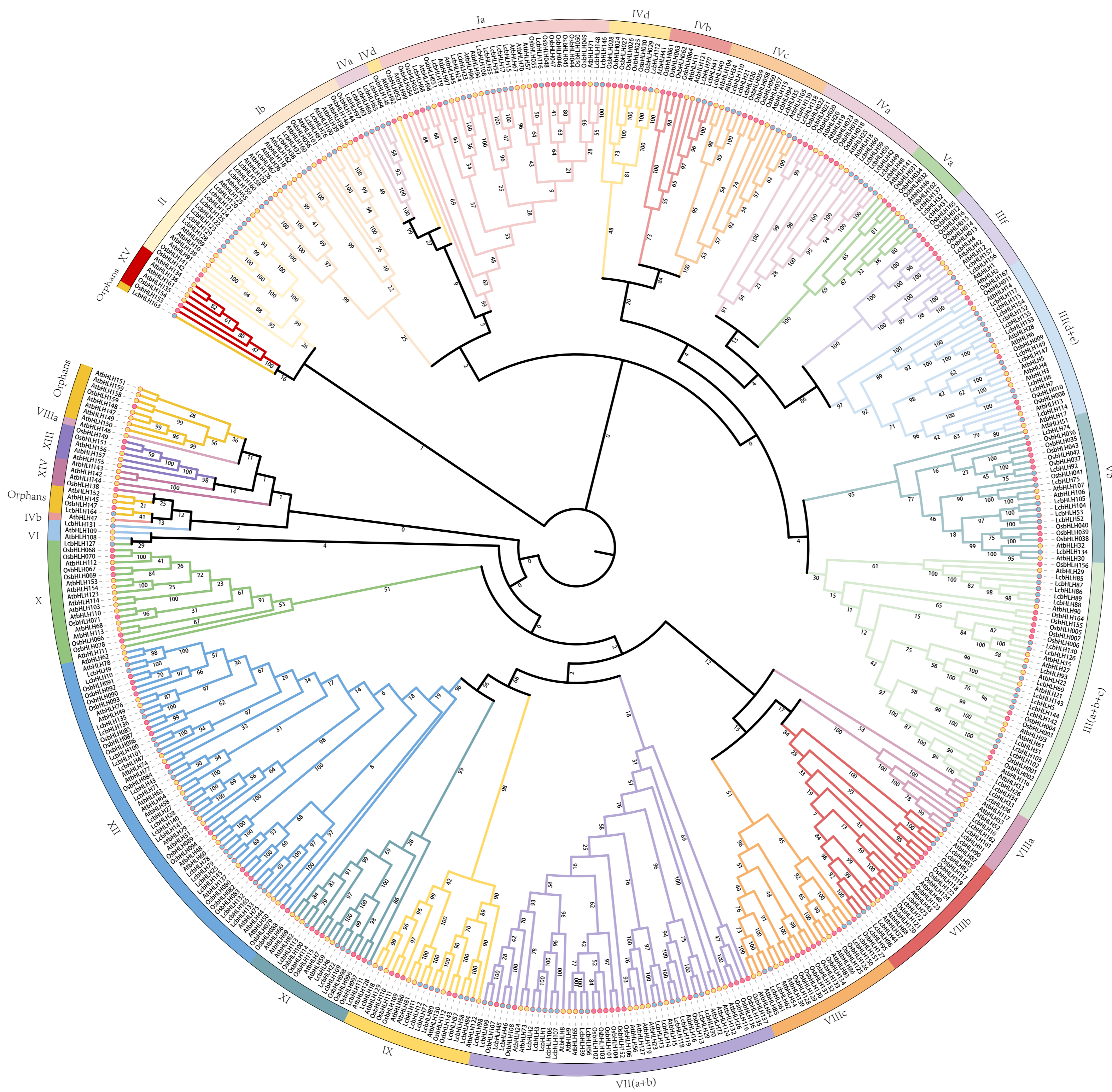

Supplement: Supplementary file 1 [file plants-12-03392-s001.zip › Figure.S1 Evolutionary tree constructed with the sequences of the LcbHLHs, OsbHLHs, and AtbHLHs.pdf]

A

B

C

D

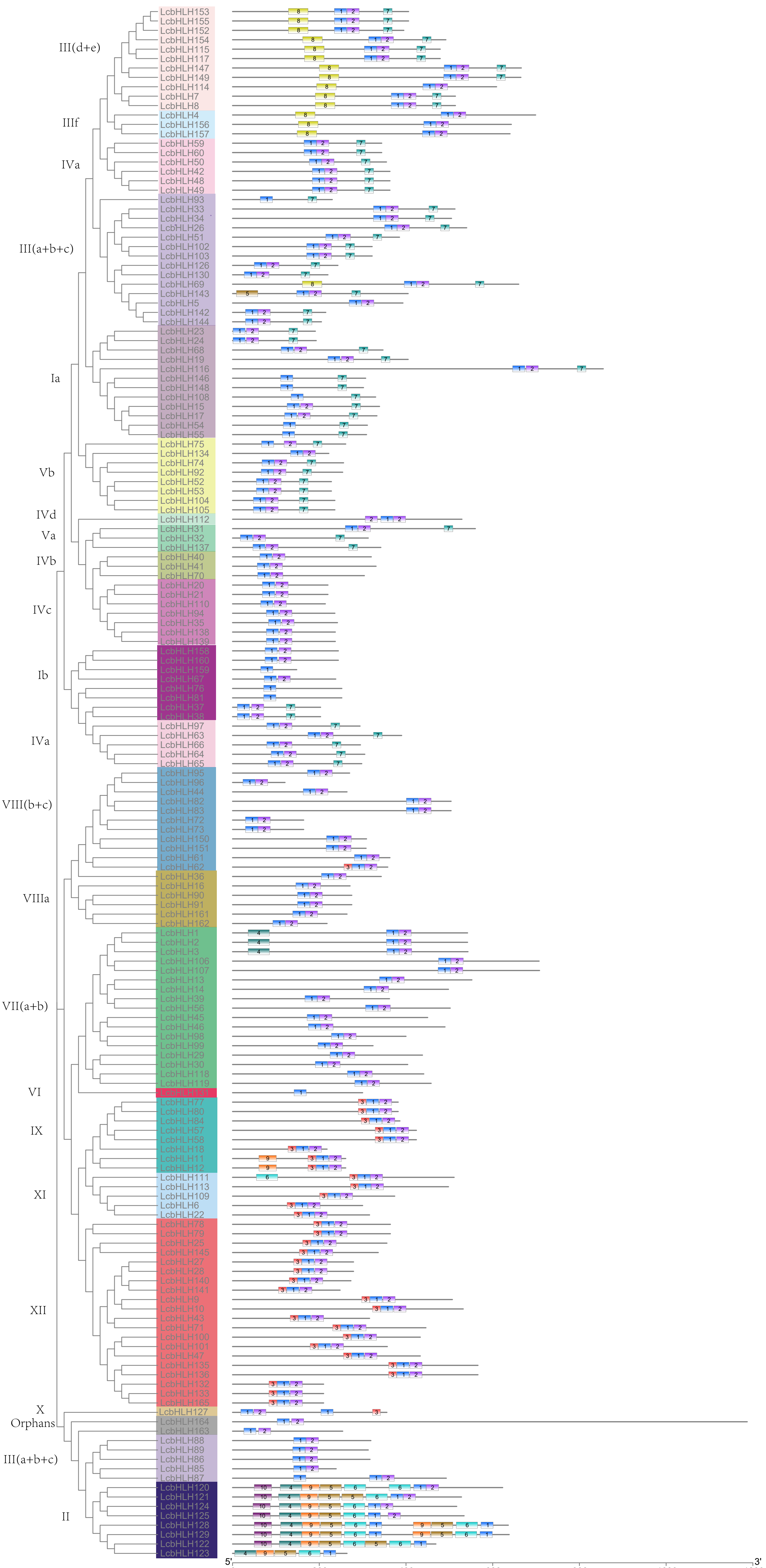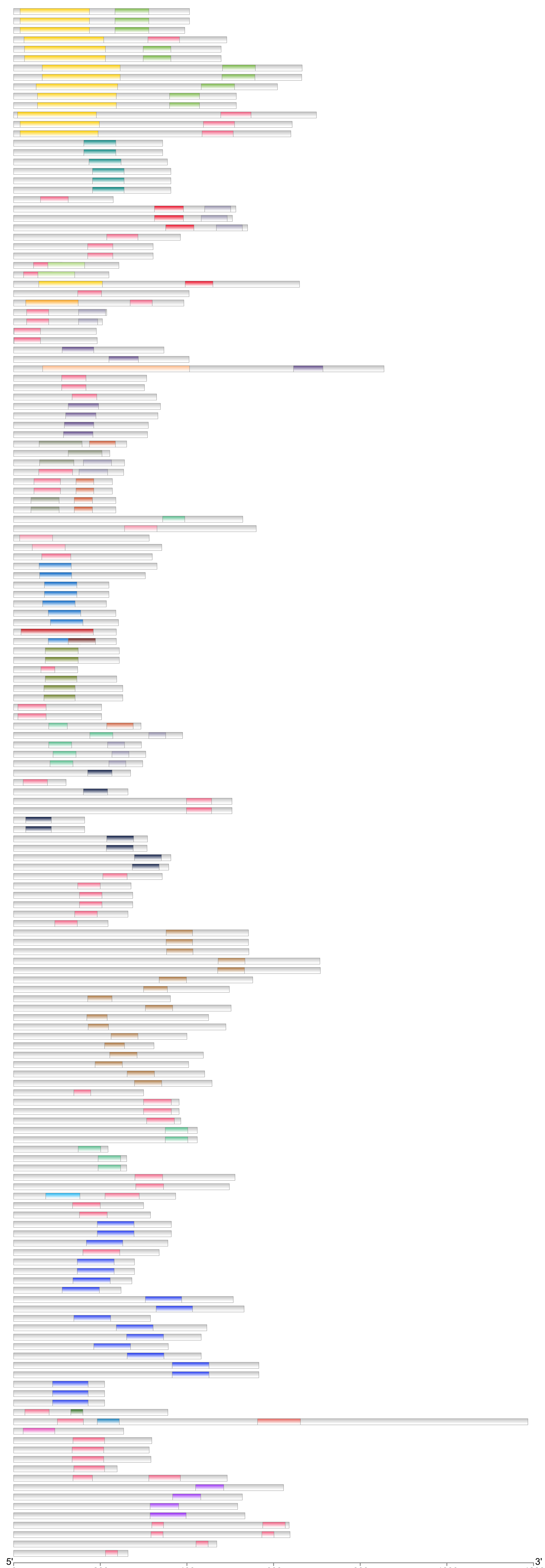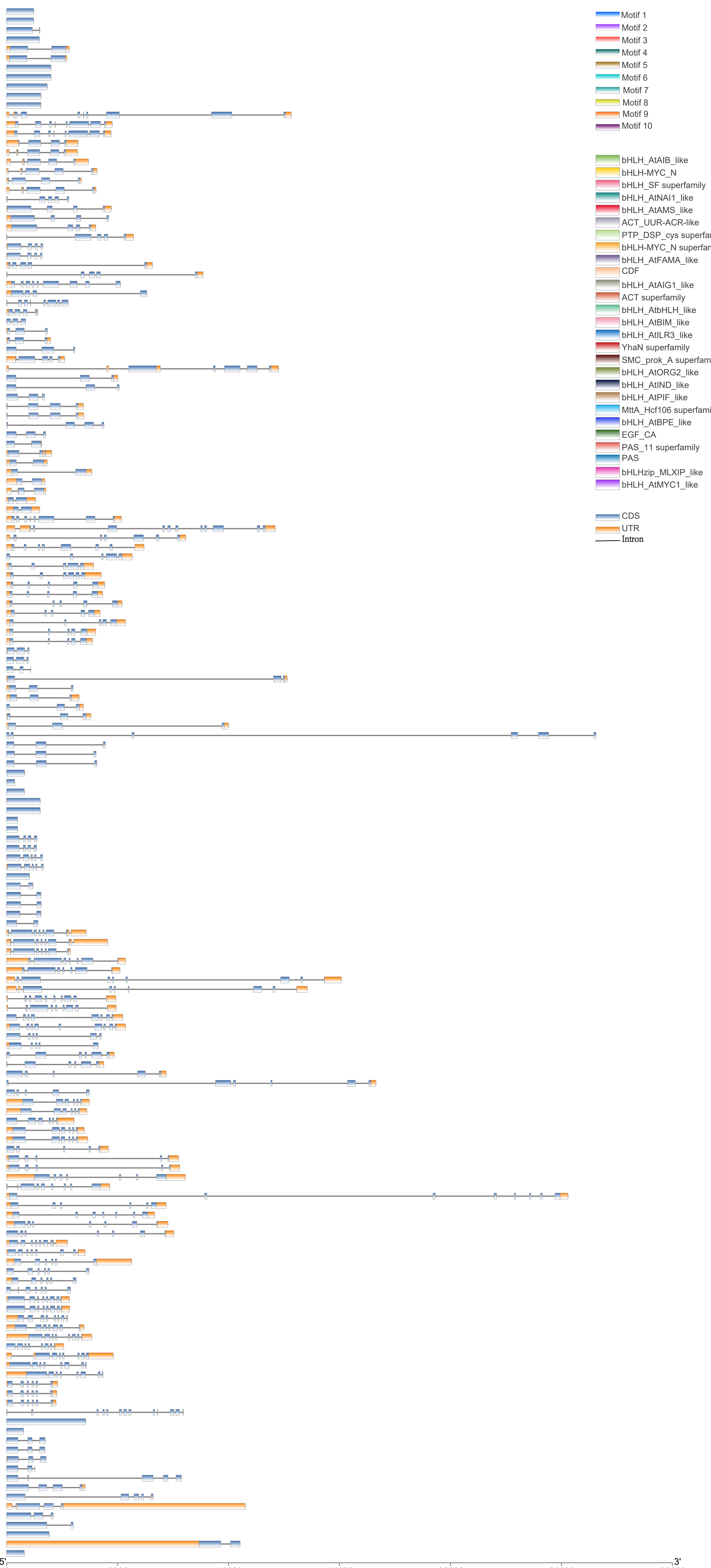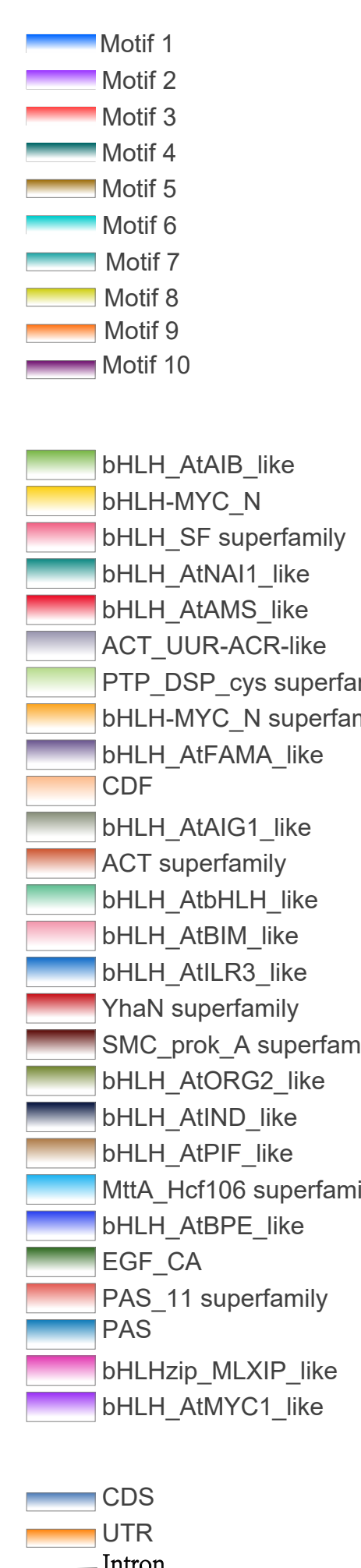

Supplement: Supplementary file 1 [file plants-12-03392-s001.zip › Figure.S2 Evolutionary relationships, conserved motifs, families of structural domains, and gene structure of the LcbHLHs.pdf]
